# Long-term Immunity Against Hepatitis B Virus After Routine Immunization Among Adults Visiting Primary Care Centers in Riyadh, Saudi Arabia

**DOI:** 10.7759/cureus.21266

**Published:** 2022-01-15

**Authors:** Mohammed A AlAteeq, Latifa M AlEnazi, Modhi S AlShammari, Essa E AlAnazi, Fadel H Al-Hababi, Abdulrahman M Alateeq

**Affiliations:** 1 Family Medicine Department, Ministry of National Guard - Health Affairs, King Abdullah International Medical Research Center, Riyadh, SAU; 2 Academy for Postgraduate Studies in Family Medicine, King Saud Medical City, Ministry of Health, Riyadh, SAU; 3 Family Medicine Residency Training Program, Ministry of Health, AlJouf, SAU; 4 Microbiology Department, Regional Laboratory, Ministry of Health, Riyadh, SAU; 5 College of Medicine, King Saud Bin Abdulaziz University for Health Sciences, Riyadh, SAU

**Keywords:** virology, prevention, immunization, infectious disease, liver

## Abstract

Objective

This study aimed to determine the persistence of induced immunity against hepatitis B virus (HBV) among adults routinely vaccinated during their infancy and correlate the level of induced immunity with participant characteristics.

Methodology

This was a cross-sectional study conducted among visitors to primary care centers of the Ministry of Health (MOH) in Riyadh, the Kingdom of Saudi Arabia (KSA) during the period from August 2020 to January 2021. The study population included healthy adults of both genders who had received full doses of the HBV vaccine in infancy. Data related to participant characteristics were collected using a self-administered questionnaire. A blood sample was then taken from each participant to measure the serum level of hepatitis B surface antigen (HBsAg), antibodies against HBsAg (anti-HBs), and antibodies against hepatitis B core antigen (ani-HBc).

Results

A total of 400 subjects participated in the study; the mean age of the cohort was 25 years. Almost all of them were Saudis (99.30%), and more than half (57.50%) were males. Only 24.30% had an anti-HBs antibodies level of ≥10 IU/L, and all respondents were negative for HBs antigen. No significant association between participant characteristics and anti-HBs antibody levels was found.

Conclusion

A decline in immunity many years after HBV vaccinations taken in infancy has been well-documented. However, for low-risk populations, the boosting of HBV vaccines is probably unnecessary since the immune memory provides sufficient protection despite low or undetectable anti-HBs antibodies.

## Introduction

Hepatitis B virus (HBV) is a DNA virus with a small, double-stranded structure and eight genotypes with variable geographic distribution for each type [1]. HBV infection is a major health issue worldwide. According to the World Health Organization (WHO), 257 million people were estimated to have chronic HBV infection in 2015 worldwide [2]. HBV infections result in significant morbidity and mortality, which are mainly related to chronic liver diseases, such as liver cirrhosis, liver failure, and hepatocellular carcinoma (HCC) [1]. The safe and effective vaccine against HBV infection has yielded good control of HBV infection, and hence, has led to a significant reduction of the associated morbidities and mortality rates.

The Kingdom of Saudi Arabia (KSA) is considered an endemic country for HBV infection in the Middle East, with an estimated prevalence of 1.3-2% [3,4]. The mandatory infant vaccination programs in the country have successfully reduced the prevalence of HBV infection from its high prevalence, which ranged between 5 and 10% 40 years ago [4]. However, the incidence of HBV infections is still considered high. The average annual incidence of HBV seropositivity is 104.6 per 100,000 population [5]. WHO recommends a dose of the HBV vaccine to be administered within 24 hours of birth to every infant to prevent perinatal HBV transmission. This birth dose should be followed by two or three additional doses [6].

In KSA, the vaccination program against HBV was initiated in October 1989 for all infants at an interval of zero, three, and five months of birth [7]. In 1990 and 1996, subsequent HBV vaccination programs were initiated by the government in KSA to cover any unvaccinated children at the time of school entry. These nationwide programs resulted in 99% protection that continued for eight years [3].

A concentration of ≥10 mIU/mL of the antibody to hepatitis B surface antigen (anti-HBs) measured one to three months after the completion of the vaccination series is regarded as a reliable measure of protection against further HBV infection [6]. Nevertheless, very little is known regarding the length of effective protection provided by HBV vaccination. This lack of understanding may represent a dilemma for clinicians as to whether a booster dose is needed or not [8]. The anti-HBs concentrations decline quickly after the first year of initial vaccination, and slowly thereafter. In 15-50% of fully vaccinated children, the anti-HBs concentrations may be low or undetectable within 5-15 years. In adults, the anti-HBs concentrations will fall below <10 mIU/mL within 9-11 years in 30-60% of the population [9].

In a cohort study with a 30-year follow-up period, Bruce et al. found that no significant infections were diagnosed in vaccinated people during the 30-year period of follow-up after the initial vaccination, and 51% of them still had anti-HBs levels ≥10 mIU/mL [10]. Other studies have observed that primary HBV vaccination can prevent infection for more than 25 years, despite the decline or loss of vaccine-induced anti-HBs antibodies over time [11-15]. Gilca et al. have reported high seroprotection rates of ≥10 IU/l in 88.2%, 86.4%, and 76.7% of Canadians at five, 10, and 15 years post-vaccination, respectively [16]. A low seroprotection rate was detected one-year post-vaccination in 65% of children in a study carried out in Iran, which declined significantly over time to 24% by 15 years of vaccination administration [17]. In KSA, Alfaleh et al. observed that only 38% of the studied cohort (16-18-year olds) retained protective anti-HBs antibodies post-vaccination [8]. In a study from Egypt, the seroprotection rate was 60.7%, and it was significantly higher among children aged <5 years compared to older children [18].

This study aimed to determine the persistence of induced immunity against HBV among adults routinely vaccinated during infancy and correlate the level of induced immunity with participant characteristics.

## Materials and methods

This was a cross-sectional, community-based study conducted among visitors to primary care centers in Riyadh, KSA during the period from August 2020 to January 2021. The study population included healthy adults of both genders who were born after 1989, when the routine HBV vaccination was started, and who had received full doses of the vaccine. Persons with chronic HBV infection, those who had received booster doses of the HBV vaccine, and those who were incapable of independent communication were excluded from the study.

The sample size was calculated based on a study by Alfaleh et al., in which it was found that 38% of the studied cohort retained protective anti-HBs post-vaccination [7]. Using a 95% confidence interval (CI) and a 5% margin of error, the required sample size was estimated to be 362. The sample size was calculated using the free online OpenEpi epidemiologic calculator. The sample size was increased to 400 to ensure adequate data. A convenient sampling technique was undertaken.

Data were collected in two stages. In stage one, participant characteristics were documented using a self-administered questionnaire. The questionnaire included questions about age, gender. nationality, history of HBV infection, and the presence of comorbidities, namely hypertension, diabetes mellitus (DM), renal disease, and dyslipidemia. In stage two, a blood sample was taken from each participant to measure the serum level of hepatitis B surface antigen (HBsAg), anti-HBs, the antibody against hepatitis B core antigen (ani-HBc). Persistent seroprotection was defined as the presence of 10 IU/L or more of anti-HBs without the presence of HBsAg or anti-HBc.

The SPSS Statistics software (IBM, Armonk, NY) was used for data entry and analysis. Numeric presentation in the form of percentages and frequencies was employed for categorical variables, whereas mean and standard deviation was used for quantitative variables. Statistical analysis was performed using the chi-square test for categorical variables and the t-test or one-way analysis of variance (ANOVA) test for the qualitative variable as appropriate; a p-value <0.05 was considered statistically significant.

Ethical approval was obtained from the Institutional Review Board at King Saud Medical City, Riyadh, KSA. Informed consent was obtained from all subjects. All information was kept confidential and used for research purposes only.

## Results

A total of 400 subjects participated in the current study, and the mean age of the cohort was 25 ±3 years. Almost all of them were Saudis (99.30%), and more than half (57.50%) were males. Only four participants (1%) reported having DM, and none of them had a history of HBV infection (Table 1).

**Table 1:** Participant characteristics (n=400) DM: diabetes mellitus

Variables		Values	%
Age, years	Minimum	18	-
	Maximum	28	-
	Mean	25	-
Gender, n	Male	230	57.50%
	Female	170	42.50%
Nationality, n	Saudi	397	99.25%
	Non-Saudi	3	0.75%
History of hepatitis infection, n	Yes	0	0.00%
	No	400	100.00%
Chronic disease, n	Yes: DM	4	1.00%
	No	396	99.00%

The results of the current study revealed that only 24.30% had an anti-HBs level of ≥10 IU/L. Besides, all respondents were negative for HBs antigen. Regarding anti-HBc levels, 99.80% were negative, and only one patient showed a positive anti-HBc result. The data are shown in Table 2.

**Table 2:** HBV serology (n=400)

Variables		N	%
Anti-HBs	<10 IU/L	303	75.75%
	≥10 IU/L	97	24.25%
HBs antigen	Positive	0	0.00%
	Negative	400	100.00%
Anti-HBc	Positive	1	0.25%
	Negative	399	99.75%

The percentage of males who showed anti-HBs level ≥10 IU/L was higher than that of females (26.10% vs. 21.80%), but this was not statistically significant (p=0.346). In addition, there was no significant association between the age of the participants and the level of anti-HBs (p=0.759). Table 3 presents the level of seroprotection anti-HBs antibodies among the participants.

**Table 3:** Seroprotective anti-HBs antibody levels (IU/L) (n=97)

Anti-HBs antibody levels	N	%
10–<50	62	64.00%
50–<100	8	8.25%
100–<500	20	20.60%
500–<1000	5	5.15%
>1000	2	20.00%
Total	97	100.00%

## Discussion

The current study findings showed that only one-quarter of the participants had persistent seroprotection after a minimum of 18 years from the time of vaccination. Several studies performed with long-term follow-up in different countries have shown that 5-15 years after vaccination in infancy, about 10%-50% of individuals had low or undetectable levels of seroprotection [19-23]. Our findings are similar to those reported in an Iranian study conducted in a similar community [17]. On the other hand, higher protection rates have been reported by some other studies. For instance, two Egyptian studies have found that about half of the studied children, aged nine months to 16 years, who completed the HBV vaccination regimen during infancy, have a seroprotection level of antibodies [18,24].

Similarly, in a cohort study conducted in the United States with a 30-year follow-up period, Bruce et al. found that 51% of subjects still had anti-HBs levels ≥10 mIU/mL [10]. Moreover, Gilca et al, in a study from Canada, reported a higher prevalence of seroprotection rates as they found that 76.7% of cases had a level ≥10 IU/l at 15 years post-vaccination [16]. A study from Thailand has found that 64% of the studied subjects have anti-HBs levels ≥10 mIU/mL 20 years post-vaccination [11]. Italian and Chinees studies have reported similar figures [12,25]. Floreani et al. conducted an 18-year follow-up study on vaccinated healthcare workers and found that an effective seroprotection level persisted in >85% of subjects after 10 years [26].

When comparing our findings with a local study, a higher seroprotection rate (38%) was reported by Alfaleh et al. [7]. It should be noted that the age of the cohort studied by Alfaleh et al. ranged from 16 to 18 years, while in our study it was 18-28 years, which might explain the difference in results. Mahallawi, in another local study, investigated the persistence of seroprotection in 335 medical students in Al Madina, KSA, who had completed the HBV vaccination series in infancy; 51% of the studied group reported having anti-HBs levels <10 mIU/mL [27]. Overall, the wide variation between the findings of different studies in reported seroprotection levels may be attributed to the variation in the prevalence of HBV infection, the age groups of the population studied, types of vaccine, and/or natural history of the HBV infection [18].

It was concluded from several studies that the initial anti-HBs level after primary vaccination and the age at which vaccination was given play an essential role in the persistence of seroprotective antibodies [11-15,21,23]. Following the vaccination, the magnitude of anti-HBs titer will determine the duration it takes to decline [21]. Schönberger et al., in a meta-analysis, identified the determinants of long-term protection after HBV vaccination in infancy. According to their findings, maternal carrier status, lower vaccine dosage than presently recommended, and the gap between the last and the preceding dose of the primary vaccine series were considered determinants for the persistence of anti-HBs antibodies ≥10 [28]. However, in our study, the anti-HBs level was not correlated with any of the participants' demographic characteristics.

In a study by Bruce et al., no significant infections were found in vaccinated persons during the 30 years of follow-up, including in individuals with declined seroprotection [10]. This aligns with our study, where only one case of anti-HBc positivity was found. In comparison, a local study done in Asir in southern KSA, in which blood samples were drawn from 10,234 people, a seroprevalence of 0.8% and 1.3% was found among people aged less than 15 years and those aged 15-24 years, respectively. In that study, anti-HBs levels were not tested, but both groups were born after the implementation of the HBV vaccination program [29].

Many studies have observed that prevention from HBV infection can be maintained for more than 25 years after the initial HBV vaccination in spite of low or undetectable anti-HBs antibodies [8,11-15]. This finding was illustrated by a challenge test with a booster dose of HBV vaccination in which previously fully vaccinated persons developed an anamnestic response. Anamnestic responses are related to immune memory where people will develop immunity whenever exposed to HBV [10,12,13,16]. Therefore, current guidelines do not recommend measuring anti-HBs titers after the complete series of HBV vaccination nor giving a booster dose for immunocompetent individuals. It is only recommended for high-risk groups, such as hemodialysis patients, babies of HBV-infected mothers, relatives of HBV carriers, HIV-positive people, and healthcare workers [9,10,12,30].

With regard to study limitations, the sample size was relatively small. Moreover, the history of hepatitis infection and other medical history was recorded based on self-reports and may be prone to inaccuracy or recall bias.

## Conclusions

It has been well-documented that people who receive HBV vaccination in infancy experience a decline in immunity as years go by. However, for low-risk populations, the boosting of HBV vaccines is probably unnecessary since the immune memory provides sufficient protection despite low or undetectable anti-HBs antibodies. This issue is still a matter of debate. Further studies on a larger scale are needed to investigate the long-term persistence of HBV immunity and the need for and effect of booster doses of HBV vaccines in low-risk populations.
